# Analytic derivation of bacterial growth laws from a simple model of intracellular chemical dynamics

**DOI:** 10.1007/s12064-016-0227-9

**Published:** 2016-05-11

**Authors:** Parth Pratim Pandey, Sanjay Jain

**Affiliations:** 1 Department of Physics and Astrophysics, University of Delhi, Delhi, 110007 India; 2 The Simons Center for Systems Biology, Institute of Advanced Study, Princeton, NJ 08540 USA; 3 Santa Fe Institute, 1399, Hyde Park Road, Santa Fe, NM 87501 USA

**Keywords:** Bacterial growth laws, Growth rate optimization, Cellular economy, Chemical dynamics, Mathematical modeling

## Abstract

Experiments have found that the growth rate and certain other macroscopic properties of bacterial cells in steady-state cultures depend upon the medium in a surprisingly simple manner; these dependencies are referred to as ‘growth laws’. Here we construct a dynamical model of interacting intracellular populations to understand some of the growth laws. The model has only three population variables: an amino acid pool, a pool of enzymes that transport an external nutrient and produce the amino acids, and ribosomes that catalyze their own and the enzymes’ production from the amino acids. We assume that the cell allocates its resources between the enzyme sector and the ribosomal sector to maximize its growth rate. We show that the empirical growth laws follow from this assumption and derive analytic expressions for the phenomenological parameters in terms of the more basic model parameters. Interestingly, the maximization of the growth rate of the cell as a whole implies that the cell allocates resources to the enzyme and ribosomal sectors in inverse proportion to their respective ‘efficiencies’. The work introduces a mathematical scheme in which the cellular growth rate can be explicitly determined and shows that two large parameters, the number of amino acid residues per enzyme and per ribosome, are useful for making approximations.

## Introduction

Bacterial cells contain thousands of molecular species and are exceedingly complex, yet they exhibit certain remarkable regularities at the system level which have been quantified experimentally. The regularities of concern in this paper are a subset of the so-called ‘bacterial growth laws’ (Monod 1949; Schaechter et al. 1958; Maaloe and Kjeldgaard 1966; Maaløe 1979; Bremer and Dennis 1996; Scott et al. 2010) which highlight the relationships between macroscopically measured quantities such as cell composition, size, growth rate and the environment or medium in which the cell grows. The empirical relationships are summarized in terms of phenomenological equations. In this paper we attempt to deduce these phenomenological relationships from a mathematical model of a cell containing a few interacting (pools of) molecular species. The population dynamics of these molecular species based on standard chemical kinetics, together with an optimization principle, gives rise to the growth laws.

When genetically identical bacterial cells drawn from an overnight culture are introduced into a vessel containing a medium with a certain concentration of nutrients, temperature, etc., they exhibit several phases of growth (Monod 1949). These include, in sequence, a lag phase where there is very little growth in the number of cells, an acceleration phase where growth picks up, an exponential phase in which the population of cells grows exponentially with time (at a constant growth rate), a deceleration phase with declining growth rate that sets in when the food begins to run out and a stationary phase where the population is constant, followed by an eventual population decline. Regularities are most apparent in the exponential phase which is often referred to as a steady state. In this phase the averages and distributions (across the population of cells) of cell doubling time, cell size at birth, intracellular concentration of ribosome, total protein and metabolites become constant in time (for as long as the exponential phase lasts). These constant average values depend upon the strain of bacteria and on the medium (its concentration of nutrients, temperature, etc.). Repeated experiments with the same strain and medium but with different initial conditions (corresponding to different overnight cultures) yield the same growth rate in the steady state and the same values of these averages. The growth laws are statements of how the growth rate and these averages depend upon the environment and cellular parameters. The first of these, due to Monod (1949), is the hyperbolic dependence of the steady-state growth rate $\mu$ upon the concentration [*F*] of a growth-limiting nutrient (or food molecule) in the medium:

1 $$\begin{aligned} \mu = \mu _\infty {\frac{[F]}{C_1 + [F]}}. \end{aligned}$$

$\mu _\infty$ is the maximum value of the growth rate possible in the medium and $C_1$ the value of [*F*] at which the growth rate is half its maximum value.

In the cell, the ribosome which assembles amino acids to produce proteins from a messenger RNA template is an important catalyst of cell growth. The amount of cellular investment in ribosomes is found to depend upon the growth rate in a characteristic manner. In particular, the ratio of ribosomal protein in the cell to total protein in the cell (by weight), referred to as the ‘ribosomal fraction’ $\Phi _R$, is found to be a linear increasing function of $\mu$ when $\mu$ is increased by improving the nutritional quality of the medium (Schaechter et al. 1958; Maaløe 1979; Bremer and Dennis 1996):

2 $$\begin{aligned} \Phi _R=\Phi _R^\mathrm{min} + {\mu \over \kappa _t}, \end{aligned}$$

where $\Phi _R^\mathrm{min}$ and $\kappa _t$ are constants. However, when $\mu$ is altered by changing the catalytic efficiency of ribosomes (e.g., by producing mutants with different catalytic efficiencies or by adding antibiotics in the medium that particularly affect the catalytic efficiency) keeping the nutritional quality of the medium the same, then $\Phi _R$ is found to be a linear decreasing function of $\mu$ (Scott et al. 2010):

3 $$\begin{aligned} \Phi _R=\Phi _R^\mathrm{max} - {\mu \over \kappa _n}, \end{aligned}$$

where $\Phi _R^\mathrm{max}$ and $\kappa _n$ are constants. The above three equations can be considered to be phenomenological equations describing bacterial growth steady states, with the six constants $\mu _\infty,C_1,\Phi _R^\mathrm{min},\Phi _R^\mathrm{max},\kappa_t ,\kappa _n$ as phenomenological constants (Scott et al. 2010). The simplicity and universality of these phenomenological laws are surprising given the complexity and diversity of bacteria. In addition to the above growth laws, the size of bacterial cells also exhibits remarkable properties which are not the subject of this paper.

There have been several recent works which have attempted to understand the growth laws theoretically, through mathematical modeling (Molenaar et al. 2009; Scott et al. 2010, 2014; Maitra and Dill 2015; Weiße et al. 2015; Bosdriesz et al. 2015). Scott et al. (2010, 2014) have related the phenomenological constants to molecular parameters of the cell. Taking forward an idea due to Maaløe (1979), they have argued that the growth laws reflect regulatory mechanisms in the cell that optimize its growth rate in any given medium. They and other authors (Maitra and Dill 2015; Weiße et al. 2015; Bosdriesz et al. 2015) have constructed models for the molecular regulatory mechanisms inside the cell that can produce the above growth laws.

In this paper we adopt a different approach that is closer in spirit to the work of Molenaar et al. (2009). Molenaar et al. considered a nonlinear dynamical model of a cell with a few classes of metabolites and enzymes as well as ribosomes and showed through computer simulations that maximization of the cellular growth rate qualitatively reproduced some of the growth laws and other observed properties of cells. Here we consider a simpler nonlinear dynamical model of the cell containing only three molecular populations: one metabolite pool, one enzyme pool and ribosomes. We are able to obtain an explicit formula for the growth rate of the cell as a function of cellular and medium parameters, which has so far been lacking in existing models. Maximizing the growth rate with respect to one of the parameters, the fraction of ribosomes making ribosomes, we derive all the three growth laws analytically. The method produces analytic expressions for the phenomenological parameters in terms of the molecular parameters in the model. These expressions are generalizations of the ones obtained by Scott et al. and reduce to their results when certain processes are ignored. We show that the optimization of growth rate leads to a simple principle of cellular economy. The work provides a direct connection between growth rate optimization and the growth laws.

At a methodological level we identify natural large parameters in the cell that are useful in making approximations. This might prove useful in more complex cellular models and in modeling other cellular phenomena as well.

## Precursor-Transporter-Ribosome (PTR) cell: a coarse grained model

**Fig. 1:**
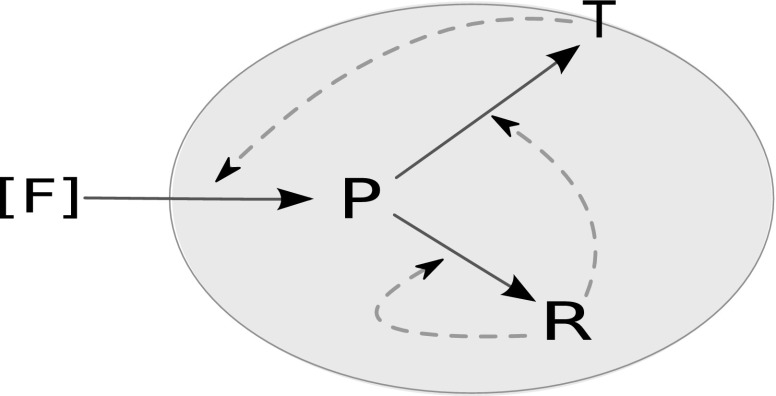
The PTR cell. Precursor molecules (*P*) are produced by the catalytic action of the metabolic proteins (*T*) on the external food molecules (*F*). Metabolic proteins and ribosomal proteins (*R*) are synthesized from the *P* molecules in reactions catalysed by *R*

Consider a simple mathematical model of a growing cell consisting of three types of molecules; precursors, transporters and ribosomes. We refer to this model as the Precursor-Transporter-Ribosome (PTR) model. The system has the following three reactions (Fig. 1):

1. $F \xrightarrow {T} P$, where external food molecules (*F*) are transported into the cell by the action of transporter proteins (*T*) and converted into precursor molecules (*P*) representing amino acids;
2. $P \xrightarrow {R} T$, where *P* molecules are converted into *T* by the catalytic action of ribosomes (*R*), and
3. $P \xrightarrow {R} R$, where *R* catalyses the production of itself using *P*.

All the molecules are produced in the interior of the cell. The membrane consists solely of transporter molecules, which are assumed to migrate immediately upon formation to the cell boundary. The interior of the cell consists of precursor molecules and ribosomes. The model is described by the following set of differential equations:

4a $$\begin{aligned} \frac{{\rm d}P}{{\rm d}t}&= K_{P}T - k\frac{RP}{V},\end{aligned}$$

4b $$\begin{aligned} \frac{{\rm d}T}{{\rm d}t}&= K_T\frac{RP}{V} - d_T T,\end{aligned}$$

4c $$\begin{aligned} \frac{{\rm d}R}{{\rm d}t}&= K_R\frac{RP}{V} - d_R R, \end{aligned}$$

where *P* represents the number of precursor molecules in the cell (amino acid pool), *T* the number of all metabolic protein molecules that transport food into the cell and convert it into precursor and *R* is number of ribosomes in the cell. The rate constant $K_P$ represents the efficiency of metabolism in making *P* from external food. It is an increasing function of the external food concentration [*F*] (explicit forms to be discussed later) and can also encapsulate the quality of the food source (e.g., the number of *P* molecules produced per food molecule transported in). The other production rate constants are parametrized as follows:

5 $$\begin{aligned} K_T = \frac{f_T k}{m_T}, \quad K_R = \frac{f_R k}{m_R}, \quad f_T + f_R = 1, \end{aligned}$$

where *k* represents ribosomal catalytic efficiency and is the rate at which a single ribosome consumes *P* molecules, per unit concentration of *P*, for the production of proteins. This accounts for the term *kRP*/*V* in the $\dot{P}$ equation, the total rate of consumption of *P*. A fraction $f_T$ of the ribosomes makes the *T* proteins and the remaining fraction $f_R$ the ribosomal proteins. Thus, of the *P* consumption flux a part $f_T kRP/V$ goes to produce *T* and the remaining part $f_R kRP/V$ goes to produce *R*. Each *T* molecule (ribosome) contains $m_T$ ($m_R$) amino acid residues; hence the rate of production of *T* is $f_T kRP/{V m_T}$ and that of *R* is $f_R kRP/{V m_R}$. This explains the assumed forms of $K_T$ and $K_R$. $d_T$ and $d_R$ are the degradation rates of *T* and *R*, respectively, into a waste product; we assume a negligible degradation rate for *P*.

*V* is the instantaneous volume of the interior of the cell and we assume that it is a linear function of the molecular populations. Since molecular populations in the bulk are *P* and *R*, we can take it to be proportional to $P+R$. Our results do not depend upon this particular choice and for generality we assume

6 $$\begin{aligned} V = v_P P + v_T T + v_R R, \end{aligned}$$

where $v_P,v_T,v_R$ are constants. Note that Eqs. (4a)–(4c) do not contain a term proportional to $\dot{V}/V$ on the right-hand side because they refer to populations instead of concentrations.

## Steady-state solution of the PTR cell

The steady state of a bacterial culture corresponds to cells growing exponentially with a constant rate. We look for an exponential solution for the chemical populations: $P(t)=P_0e^{\mu t}, T(t)=T_0e^{\mu t}, R(t)=R_0e^{\mu t}$, where $\mu$, a constant, is the growth rate of the PTR cell. Substituting this ansatz into Eq. (4), we get

7a $$\begin{aligned} \mu P_0&= K_{P}T_0 - k \frac{R_0P_0}{V_0}, \end{aligned}$$

7b $$\begin{aligned} (\mu + d_T) T_0&= K_T \frac{R_0P_0}{V_0}, \end{aligned}$$

7c $$\begin{aligned} (\mu + d_R) R_0&= K_R \frac{R_0P_0}{V_0}, \end{aligned}$$

where $V_0 = v_PP_0 + v_TT_0 + v_RR_0$. Henceforth we drop the subscript 0 as the equations are valid for the time-dependent quantities *P*(*t*), *T*(*t*), *R*(*t*) as well. The last of these equations immediately gives

8 $$\begin{aligned} P/V=(\mu + d_R)/K_R. \end{aligned}$$

Substituting (8) in (7b) gives the ratio *T*/*R*:

9 $$\begin{aligned} \frac{T}{R}=\frac{m_R}{m_T}\frac{f_T}{f_R}\frac{(\mu + d_R)}{(\mu + d_T)}, \end{aligned}$$

and substituting (8) and (9) in (7a) gives the ratio *P*/*R*:

10 $$\begin{aligned} \frac{P}{R} = \frac{m_R}{f_R}\frac{(\mu + d_R)}{\mu }\left( \frac{K_P f_T}{m_T(\mu + d_T)} - 1 \right) . \end{aligned}$$

Thus the ratios of the populations and the concentrations of the three chemicals at steady state can be expressed in terms of $\mu$ and the parameters of the model. In order to solve the problem fully, we need to find $\mu$ in terms of the parameters.

*Growth rate*   The Eq. (8) gives $\mu = K_R P/V - d_R$. Note that *V* can be written as $V=v_P P(1 + \frac{v_T}{v_P}\frac{T}{P} + \frac{v_R}{v_P}\frac{R}{P}) = v_P P(1 + [\frac{v_T}{v_P}\frac{T}{R} + \frac{v_R}{v_P}]\frac{R}{P})$. Thus *P*/*V* is completely expressed in terms of the ratios *T*/*R* and *P*/*R* which are known as functions of $\mu$ and the parameters [Eqs. (9) and (10)]. Therefore, the equation $\mu = K_R P/V - d_R$ becomes an equation that contains only $\mu$ and the parameters. Simplifying it, we get a quadratic equation in $\mu$ with coefficients depending on the parameters:

11 $$\begin{aligned} \alpha \mu ^2 - \beta \mu + \gamma = 0 \end{aligned}$$

with

12a $$\begin{aligned} \alpha&= 1 - \epsilon _1, \quad \beta = a + b + \epsilon _2, \quad \gamma = ab;\end{aligned}$$

12b $$\begin{aligned} a&= \nu f_T - d_T, \quad b = \rho f_R - d_R;\end{aligned}$$

12c $$\begin{aligned} \nu&= K_P/m_T = \mathrm{{`{nutritional\,efficiency}'}}, \end{aligned}$$

12d $$\begin{aligned} \rho&= k/(m_R v_P) = \mathrm{{`{ribosomal\,efficiency}'}};\end{aligned}$$

12e $$\begin{aligned} \epsilon _1&= \frac{1}{m_T} \frac{v_T}{v_P} f_T + \frac{1}{m_R}\frac{v_R}{v_P} f_R,\end{aligned}$$

12f $$\begin{aligned} \epsilon _2&= \frac{1}{m_T}\frac{v_T}{v_P} f_T d_R + \frac{1}{m_R}\frac{v_R}{v_P} f_R d_T. \end{aligned}$$

Equation (11) has two solutions:

13 $$\begin{aligned} \mu _{\pm } = \frac{\beta \pm \sqrt{ \beta ^{2} - 4\alpha \gamma }}{2\alpha }. \end{aligned}$$

The $\mu _-$ solution is the physically relevant one, in which the square-root is always taken with the negative sign. There are several ways to see this:

1. The Eqs. (4a)–(4c) can be simulated numerically for a fixed set of parameter values and initial conditions. This was done for several parameter sets and initial conditions. We found that at large times *P*, *T* and *R* always grew exponentially with time and their rate of exponential growth was given by $\mu _-$ and not $\mu _+$. Further, the observed asymptotic ratios were given by Eqs. (9), (10) with $\mu =\mu _-$. (Parameter values had to be chosen such that $\mu _- >0$. When parameter values were such that $\mu _- < 0$, an exponential decline of populations was observed instead of growth.)
2. One can examine the two limits $f_R \rightarrow 0$ and $f_R \rightarrow 1$. When $d_T=d_R=0$, in both these limits $\mu$ must go to zero. Physically, when $f_R \rightarrow 0$, then $K_R \rightarrow 0$ and Eq. (4c) implies that ribosomes are not produced; hence *R* is a constant, or $\mu =0$. When $f_R \rightarrow 1$, then $K_T \rightarrow 0$, and *T* is not produced; hence again $\mu =0$. It is easy to see that $\mu _-$ goes to zero in both these limits and not $\mu _+$.
3. We have verified analytically from Eq. (7) that when $d_T=d_R=0$ and $m_T=m_R \gg 1$, $\mu _+$ gives rise to negative populations while $\mu _-$ gives rise to positive populations.

We remark here that it has been possible to obtain an explicit solution for $\mu$ because we have expressed the cell volume as a function of the populations and further assumed that it is a linear function of the populations, (6). This assumption (a) makes the exponential ansatz a solution of (4), and (b) causes the absolute populations to be eliminated from (8), leaving an equation connecting $\mu$ and the parameters. In our view the volume assumption is a crucial one that has been missing from previous models.

*Ribosomal fraction* ($\Phi _R$)  The ratio of ribosomal protein to total protein (by weight) is given by $\Phi _R = \displaystyle \frac{m_RR}{m_TT + m_RR}$. Using Eq. (9) $\Phi _R$ becomes

14 $$\begin{aligned} \Phi _R= & {} \frac{1}{1 + \displaystyle \frac{f_T}{f_R}\frac{(\mu +d_R)}{(\mu +d_T)}} = f_R + \frac{f_Tf_R(d_T - d_R)}{\mu + f_Td_R + f_Rd_T}. \end{aligned}$$

Notice that this expression for $\Phi _R$ is a nonlinear function of $\mu$ if $d_T \ne d_R$ and a constant independent of $\mu$ if $d_T=d_R$. This is quite different from the observed linear growth laws (2) and (3). Thus the PTR model does *not* reproduce the observed growth laws. The model as it stands is missing an important ingredient—regulation—that we now turn to.

## The PTR model with ‘regulation’ and bacterial growth laws

Upto now we have treated $f_T$ and $f_R$, the fraction of ribosomes catalysing the production of transporters and ribosomal protein, respectively, as constant parameters of the model. However, it is a well-known fact that regulatory mechanisms exist in bacteria that regulate how much ribosome is engaged in producing ribosomal protein and how much in producing metabolic protein. In the context of the PTR model these mechanisms would modulate the value of the $f_R$ parameter (and hence $f_T=1-f_R$). The absence of this mechanism in the PTR model as described above is the reason that it does not reproduce the observed growth laws.

**Fig. 2:**
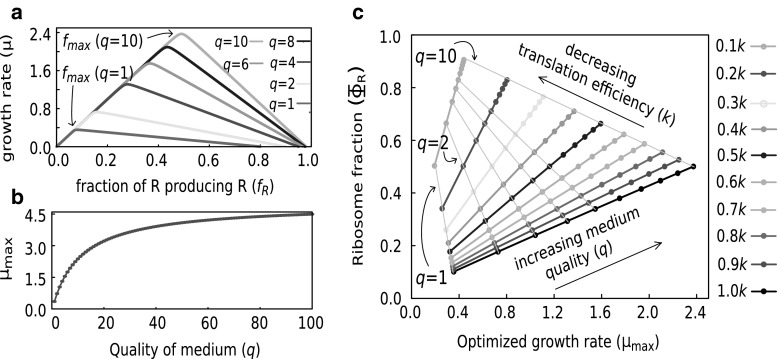
The PTR model in the optimized steady state qualitatively reproduces the observed growth laws. **a** Trade-off between production of ribosomal and metabolic proteins: $\mu$ as a function of $f_R$ for different values of *q* ($K_P=qk_P$, $k_P=250$ $hr^{-1}$, $k=5*10^{-4}$ $hr^{-1}$ ${\mu m}^{3}$, $d_T = 0.1$ $hr^{-1}$, $d_R=0$ $hr^{-1}$, $m_R = 10^4$, $m_T = 5*10^2$, $v_P=v_T=v_R=10^{-8}$ ${\mu m}^{3}$). **b** $\mu _{\mathrm{max}}$ as a function of *q*. Other parameters same as in **(a)**. **c** $\Phi _R$ versus $\mu _{\mathrm{max}}$ for *different values* of *q* and *k* (other parameters same as before). The *coloured lines* (positive slope) correspond to Eq. (2) (changing medium quality at fixed translational efficiency). The *grey lines* (negative slope) correspond to (3) (changing translational efficiency at fixed medium quality)

*Trade-off between metabolic and ribosomal protein production*   Since $\mu$ is a function of the cellular and medium parameters [Eq. (13)], we first ask how it varies as $f_R$ is increased keeping the medium and all other cellular parameters fixed. Numerical analysis of the steady-state of the PTR model shows that when all other parameters are fixed, $\mu$ is a non-monotonic function of $f_R$ as shown in Fig. 2a. This reflects a trade-off between production of metabolic proteins and ribosomal proteins in the model. There is a distinct value of $f_R$ ($f_\mathrm{max}$) where $\mu$ is a maximum ($\mu _\mathrm{max}$). $f_\mathrm{max}$, $\mu _\mathrm{max}$ depend upon the other parameters and in particular, $f_\mathrm{max}$ increases as $K_P$ is increased (keeping the others constant). For convenience we here write $K_P = q k_p$ where *q* equals the number of *P* molecules produced per food molecule consumed (*quality* of the medium), and $k_P$ depends upon external food concentration. We observe in Fig. 2a that as the quality of medium is increased, $f_{\mathrm{max}}$ increases. These two properties, namely non-monotonicity of $\mu$ with respect to $f_R$ and the increase of $f_{\mathrm{max}}$ with medium quality have also been noted in Scott et al. (2014) using a different approach.

*Incorporating the effect of regulatory mechanisms through an optimization assumption*  In order to bring in regulatory mechanisms we can make the rate constants dependent on molecular concentrations reflecting feedback mechanisms or introduce other molecular species (the regulators) into the model (Scott et al. 2014; Maitra and Dill 2015; Weiße et al. 2015; Bosdriesz et al. 2015). However, in the interest of mathematical simplicity we take an alternative approach involving optimization, employed earlier by Molenaar et al. (2009) for a different model. We assume that for any fixed medium and other cellular parameters additional regulatory mechanisms existing in the cell act to modify $f_R$ (e.g., by changing the proportion of messenger RNA molecules corresponding to *R* and *T*) such that the cellular growth rate is optimized, i.e., for a given medium and other cellular parameters, the regulation adjusts $f_R$ to $f_{\mathrm{max}}$. This is in spirit similar to the optimality assumption made in flux balance analysis of metabolic networks (Orth et al. 2010). In other words, we assume that the steady state reached when these other (unspecified) regulatory dynamics are included is approximated by the steady state of the PTR model with

15 $$\begin{aligned} f_R = f_\mathrm{max}, \end{aligned}$$

where $f_\mathrm{max}$ is the value of $f_R$ that maximizes $\mu _-$ [Eq. (13)] keeping all other parameters fixed. We call this steady state (when $f_R$ is set equal to $f_{\mathrm{max}}$) as the *optimized steady-state*. A change in medium, in general, leads to a different $f_{\mathrm{max}}$ since $\mu$ is a function of all the medium-dependent parameters parameters and $f_R$.

*Optimized steady state of the PTR cell reproduces qualitative features of observed growth laws*  Figure 2b, c shows that the optimized steady state of the PTR cell qualitatively satisfies the growth laws summarized in Eqs. (1)–(3). In Fig. 2b to increase the growth rate for the PTR cell we only increase the medium quality *q* (keeping $k_P,k,m_T,m_R,d_T,d_R,v_P,v_T,v_R$ constant). For each medium quality *q* we numerically obtain $f_{\mathrm{max}}(q)$ using Eq. (13), i.e., the value of $f_R$ that gives the largest $\mu$ for the given *q*. We denote this optimized $\mu$ as $\mu _{\mathrm{max}}(q)$ since it depends on *q*. We plot the dependence of $\mu _{\mathrm{max}}$ on *q* and find the qualitative behaviour similar to the Monod curve (1).

Next we show the dependence of ribosome fraction $\Phi _R$ on $\mu _{\mathrm{max}}$ in the optimized steady state when $\mu _\mathrm{max}$ is increased by increasing *q*. For each medium quality *q*, we already have $f_{\mathrm{max}}(q)$ and $\mu _{\mathrm{max}}(q)$. To obtain $\Phi _R$ we use the relation Eq. (14) with $f_R=f_{\mathrm{max}}$ and $\mu =\mu _{\mathrm{max}}$. Figure 2c shows the plot of $\Phi _R$ versus $\mu _{\mathrm{max}}$ as the quality of the medium is increased. The lines with positive slope in Fig. 2c correspond to this variation. Notice the linear behaviour of the curves as has been observed in experiments, Eq. (2).

For a smaller value of *k* (smaller ribosomal efficiency), the $\Phi _R$ versus $\mu _{\mathrm{max}}$ curve remains linear but with a larger slope (coloured lines in 2c) as has been observed in experiments (Scott et al. 2010). Figure 2c qualitatively reproduces the observed behaviour of $\Phi _R$ [Eqs. (2), (3)] when the growth rate is varied, both by increasing medium quality and by decreasing ribosomal efficiency.

*Analytic derivation of the growth laws for the PTR model—the large*  $m_T,m_R$*approximation* The above results obtained numerically and illustrated in Fig. 2 can be derived analytically. The expressions turn out to be very simple when $m_T$ and $m_R$ are much greater than unity, which we assume in the following ($m_T$ being the number of amino acid molecules needed to make an enzyme is ${\sim}300$, and $m_R$, the number of amino acids in all ribosomal protein per ribosome is ${\sim}7000$ Bremer and Dennis 1996). We also need to assume that the parameters $\nu$ and $\rho$ defined in (12) are independent of $m_T$ and $m_R$; in other words, $K_P$, the rate of *P* production per unit *T* molecule in the cell, and $k/v_P$, the rate at which a ribosome adds amino acids to a protein, are sufficiently large, in fact, respectively, of order $m_T,m_R$. $\nu$ and $\rho$ will turn out to be the two natural time scales that determine the system level properties of the cell. The time scales $d_T,d_R$, and the volume $v_P$ will also be assumed to be independent of $m_T,m_R$. $v_T,v_R$ may be independent or only weakly dependent on $m_T,m_R$, respectively (sublinear dependence). With these assumptions, $\epsilon _1 \ll 1$ and $\epsilon _2 \ll a+b$.

Then, as shown in the “Appendix”,

16 $$\begin{aligned} \boxed {f_{\mathrm{max}}={{\nu +d_R - d_T} \over {\nu +\rho }}}, \end{aligned}$$

and the optimized steady-state growth rate of the PTR cell is given by

17 $$\begin{aligned} \boxed {\mu =\mu _{\mathrm{max}}= \frac{\rho (\nu -d_T) - \nu d_R}{\nu +\rho }}. \end{aligned}$$

This leads to the Monod curve as will be discussed later.

Substituting $f_R=f_{\mathrm{max}}$ and $\mu =\mu _{\mathrm{max}}$ in Eqs. (9) and (14) gives

18 $$\begin{aligned} {T \over R}=\frac{m_R}{m_T}\frac{\rho }{\nu }, \end{aligned}$$

19 $$\begin{aligned} \boxed {\Phi _R = {\nu \over {\nu +\rho }}}. \end{aligned}$$

This expresses the ribosomal fraction at the optimized steady state of the PTR cell in terms of medium and cellular parameters. The growth laws in the standard form (2), (3) follow from (19) and (17). For example, to understand the dependence of $\Phi _R$ on $\mu$ when the medium quality is varied, one can eliminate $\nu$ in favour of $\mu$ in Eq. (17) and substitute that in Eq. (19). This yields (2) with

20 $$\begin{aligned} \Phi _R^\mathrm{min}= {d_T \over {\rho +d_T - d_R}}, \quad \kappa _t = \rho + d_T - d_R. \end{aligned}$$

Similarly, one can eliminate $\rho$ in favour of $\mu$ from Eq.  (17) and substitute in Eq. (19) to get Eq. (3), with

21 $$\begin{aligned} \Phi _R^\mathrm{max}= \frac{\nu - d_T}{\nu -d_T + d_R}, \quad \kappa _n = \nu -d_T + d_R. \end{aligned}$$

This reproduces the equations of the growth laws and expresses the constants appearing in those equations in terms of the medium and cellular parameters. Equation (2) with parameters given by (20) describes the positive slope lines in Fig. 2c and Eq. (3) with parameters given in (21) describes the negative slope lines.

## Discussion

*Nutritional and ribosomal efficiency*  We now discuss the meaning of the formulae obtained. The formulae are expressed in terms of two quantities $\nu$ and $\rho$ and it is useful to interpret these quantities first. We follow Scott et al. (2014) in calling $\nu$ the *‘nutritional efficiency’* of the PTR cell in the given medium. Note that the production term in $\dot{P}$ is $K_P T = \nu m_T T$. Since $m_T T$ is the total number *P* molecules locked up in *T*, $\nu$ is the number of amino acid molecules produced in the cell per unit time per amino acid residue locked up in the metabolic enzymes. $\nu$, being the rate of *P* production per unit *P* invested in metabolic enzymes, is appropriately the ‘metabolic efficiency’ or ‘nutritional efficiency’ of the cell in the given environment. In order to see the meaning of $\rho$ it is convenient to consider the situation where the concentration of *P* is high enough so that its availability is no longer a limiting factor for ribosomal activity. In the model the largest value of *P*/*V* is $1/v_P$, which arises when the contribution of *P* to the volume dominates over the contribution from *T* and *R*, i.e., $V \sim v_PP$. Then (4c) becomes $\dot{R}=(\rho f_R - d_R)R$. Then *R* by itself forms an autocatalytic set (ACS) with growth rate $\rho f_R - d_R$. $\rho$ is the maximal growth rate of this ACS (when $d_R=0$ and $f_R=1$), or the rate at which *R* can make copies of itself if it was solely focused on doing that (that is, if $f_R=1$). $\rho$, being the maximal rate of *R* production per unit *R* present, will be referred to as the *‘ribosomal efficiency’* of the cell. The factor $k/v_P$ in $\rho =k/(v_Pm_R)$ is the rate at which a ribosome can add an amino acid to a protein when there is no limitation of *P* and the factor of $m_R$ accounts for the number of *P* required to make a ribosome. In Scott et al. (2014) $\rho$ is referred to as the ‘translational efficiency’ of the cell.

*Optimization as a principle of cellular economy*  As mentioned earlier, the growth laws (2) and (3) follow from (19). The latter is a more basic equation as it expresses $\Phi _R$ directly in terms of the parameters without reference to the growth rate, and it encapsulates the consequence of growth rate maximization when $m_T,m_R \gg 1$. (19) or equivalently (18) can be recast as

22 $$\begin{aligned} (m_TT)\nu = (m_RR)\rho . \end{aligned}$$

We can interpret $m_TT$ as the allocation or investment of the cell in the metabolic sector (measured in units of *P*) and $m_RR$ as the investment in the ribosome sector. We define the ‘output’ of each sector as the ‘investment’ times ‘efficiency’ of the sector. Then the investment strategy of the cell, namely (22), can be stated as

23 $${\mathrm{`Output}}\text{'}{\mathrm{\;of\;metabolic\;sector}} = {{\mathrm{`Output}}\text{'}{\mathrm{\;of\;ribosomal\;sector}}}.$$

Equivalently, (22) can be stated as the following principle of cellular economy: the resources allocated to the enzyme and ribosomal sectors are inversely proportional to their respective efficiencies. In other words, the PTR cell follows the dictum: From each sector according to its ability, to each sector according to its need. Here ‘ability’ of a sector is the same as its ‘efficiency’, defined earlier, and ‘need’ is the allocation or investment in the sector that would make its ‘output’ equal to that of the other sector. This principle follows from the optimization of the growth rate of the PTR cell as a whole in the large $m_T,m_R$ approximation. Note that efficiency is hardwired into the cellular and medium parameters while the allocation, in the context of the present model, is a matter of cellular ‘choice’ (though, of course, in practice, even that is hardwired into the regulatory mechanisms that dynamically implement the ‘choice’.)

We remark that (22) is not a requirement for the system to have a steady state. Indeed, steady states are achieved in the model even when $f_R$ is not at its optimal value given by (16), as discussed earlier. When $f_R \ne f_\mathrm{max}$, we can still have a steady state with constant concentrations satisfying the Eqs. (8)–(14), but (22) does not hold. (22) is the condition that the steady state has the maximal possible value of $\mu$ given that all parameters other than $f_R$ are fixed.

*The Monod curve*   We turn to a discussion of the analytic expression for $\mu$, Eq. (17). First we discuss the situation when $d_T=d_R=0$. Then from (20), (21), $\kappa _t = \rho$ and $\kappa _n=\nu$, and our results for $\mu$ and all the other quantities reproduce exactly the results of Scott et al. (2010, 2014). The growth rate reduces to

24 $$\begin{aligned} \mu = \frac{\rho \nu }{\rho + \nu }. \end{aligned}$$

This is the same as the expression $\mu =(\Phi _R^\mathrm{max} - \Phi _R^\mathrm{min})\rho \nu /(\rho +\nu )$ derived in Scott et al. (2010, 2014), when (20) and  (21) are used to set $\Phi _R^\mathrm{min}=0,\Phi _R^\mathrm{max}=1$. To make contact with the Monod equation (1), one has to say how $\nu$ depends upon the concentration [*F*] of the external nutrient. As mentioned below (4) $K_P$ and hence $\nu$ is an increasing function of [*F*]. If one substitutes the simplest function $\nu =k_1[F]$, where $k_1$ is a constant, into (24), one obtains (1) with $\mu _\infty = \rho$ and $C_1=\rho /k_1$. Alternatively, if the transport limited Michelis-Menten form of food uptake $\nu =\nu _0 [F]/(K+[F])$, where $\nu _0$ and *K* are constants, is substituted in (24), one gets (1) with $\mu _\infty = \rho \nu _0/(\rho +\nu _0)$ and $C_1 = K/[1+(\nu _0/\rho )]$ (Scott et al. 2014).

The difference between our derivation of (24) and that of Scott et al. is that the latter uses the growth laws (2), (3) as the starting point and obtains the above mentioned expression for $\mu$. It does not require any further assumption of growth rate optimality in deriving that expression as (2), (3) already incorporate optimality. On the other hand, our derivation starts with equations (4) describing the dynamics of the three pools, obtains $\mu$ in the steady state before optimization and then uses the optimality assumption to derive (2), (3) as well as the optimized $\mu$. This crisply establishes the relationship between optimality and the growth laws.

It may be helpful to make a few remarks about (24). The right-hand side is a symmetric function of $\nu$ and $\rho$, which define the two natural time scales in the problem. (1) For fixed $\rho$ as a function of $\nu$, it saturates at a maximum value $\mu =\rho$. The saturation is not a consequence of a Michelis-Menten type saturation kinetics assumed in the model [Eq. (4) has no Michelis-Menten or Hill type terms], but is a consequence of the existence of these two time scales in cellular dynamics. When $\nu \gg \rho$, $f_R$ in (16) approaches 1; thus the core autocatalytic set that drives the PTR cell—ribosome producing more ribosome—is focused largely on producing itself. Even then, we know that the maximal rate of *R* self-reproduction production can only be $\rho$, which explains the saturation. (2) Interestingly, not only is the saturation value of $\mu$ equal to $\rho$, the value of $\nu$ at which $\mu$ is half its maximum value is also $\rho$. This has a simple explanation. If, to achieve the maximum growth rate, the ribosome pool is focused solely on making ribosome, then at half the maximal rate only half the pool is focused on making ribosome. The other half is then focused on making *T*, and this equal investment in both sectors means $m_TT=m_RR$. But from the principle of cellular economy the two sectorial outputs are equal; therefore, $\nu$ must be equal to $\rho$. An alternative way of saying this is to observe from (16) that $f_R=1/2$ at $\nu = \rho$. (3) The symmetry between $\nu$ and $\rho$ implies that if $\nu$ is held fixed and $\rho$ is increased, $\mu$ will saturate at a value $\nu$, and the value of $\rho$ at half-saturation is also $\nu$.

*Dissipation terms*   Equation (17) is a generalization of (24) when $d_T,d_R$ are nonzero. Note that even with the additional terms there is a symmetry between the two sectors: $\mu$ is unchanged under the simultaneous interchange $\nu \leftrightarrow \rho$, $d_T \leftrightarrow d_R$. When $d_R=0,d_T > 0$, the factor $\nu - d_T \propto K_P - m_Td_T$ in the numerator reflects that the metabolic efficiency has to be $> d_T$ to sustain a nonzero growth rate. This is because for every $K_P$ molecules of *P* produced by each molecule of *T* per unit time, a number $m_Td_T$ is lost through the $-d_TT$ term. Similarly, when $d_T=0$, $d_R > 0$, the factor $\rho - d_R$ in the numerator means that the ribosomal efficiency has to be greater than $d_R$ for the ribosomal ACS to get off the ground. A nonzero $d_R$ requires a greater fraction of ribosomes to be making ribosomes, and a nonzero $d_T$ requires a greater fraction to be making *T* [see Eq. (16)]. However, the relative investment by the cell in the two sectors as measured by *T*/*R* or $\Phi _R$ is independent of $d_T,d_R$ [see Eqs. (18), (19)]. The equality of $f_R$ and $\Phi _R$ has been commented upon by Scott et al. (2010). They have considered models in which the degradation terms are zero. In the present model also $f_R=\Phi _R$ when $d_T=d_R$. But when $d_T \ne d_R$, the two are not equal.

We note that in the model the phenomenological parameter $\Phi _R^\mathrm{min}$ is zero if $d_T=0$, and the $\Phi _R^\mathrm{max}=1$ if $d_R=0$ [Eqs. (20), (21)]. In bacterial cells $d_T$ may be of the order of 0.1 h$^{-1}$ (Dressaire et al. 2009; Maitra and Dill 2015), while $d_R$ may be much lower (Zundel et al. 2009). This predicts a value of $\Phi _R^\mathrm{min}$ about 2–3 times smaller than the observed value given in Scott et al. (2010). Equation (20) predicts that when $d_T > d_R$, $\kappa _t$ as a function of $\rho$ is linear with a positive intercept. This feature is seen in the data (Scott et al. 2010). However, again the value of the intercept predicted by (20) is smaller than the value from the data. This suggests that other contributions to $\Phi _R^\mathrm{min}$ and $\kappa _t$, not described by the present model, are significant.

*The ‘constant fraction’ sector*  Scott et al. (2010) introduced another sector of proteins *Q* in addition to *T* and *R* which takes up a fixed fraction of the protein mass $\Phi _Q$, to account for the fact that $\Phi _R^\mathrm{max}$ was observed in experiments to be less than unity. In the present model this sector can be added as follows (we consider the case $d_T=d_R=0$): to (4), add another equation $\dot{Q}=K_Q RP/V$, where $K_Q=f_Q k/m_Q$. The other changes are in (5), where we now have $f_T+f_R+f_Q=1$, and in (6), where a term $v_Q Q$ is added to the definition of *V*. $\Phi _R$ is now defined by $m_RR/(m_TT+m_RR+m_QQ)$. In the optimization, $f_Q$ is treated as a fixed number; $f_R$ can range between 0 and $1-f_Q$ and is chosen to maximize the growth rate. Doing the analysis as for the PTR model, one reproduces the growth laws (2) and (3) in which $\Phi _R^\mathrm{min},\kappa _t$ are the same as for the PTR model, and $\Phi _R^\mathrm{max}=1-f_Q$, $\kappa _n=\nu (1-f_Q)$. Further, $f_R=\nu (1-f_Q)/(\nu +\rho )$, and, as before, $\mu = \rho f_R$, $\Phi _R = f_R$.

*A simpler derivation of the results—from a linear model*  Above, we have presented a detailed derivation of $f_R$, $\mu$ and $\Phi _R$ from the PTR model assuming $m_T,m_R \gg 1$. It is worth mentioning that the same results follow from a much simpler heuristic argument. Supposing we assume that the dominant contribution to *V* is $v_PP$, i.e., we ignore the contribution of *T* and *R* to *V*. (This does not mean that the contribution of *T* and *R* to the mass of the cell is much smaller than that of *P*. If $m_T,m_R \gg 1$, the contribution of *T* and *R* to the mass of the cell could be large, even larger than the contribution of *P*, while their contribution to the volume is much smaller than that of *P*, as long as $v_T,v_R$ are independent of (or sufficiently weakly dependent on) $m_T,m_R$). Then (4) reduces to a set of linear equations $\dot{X} = AX$ with

25 $$\begin{aligned} X =\left( \begin{array}{l} P\\ T\\ R\\ \end{array}\right) , \quad A =\left( \begin{array}{ccc} 0 &{}\quad K_P &{}\quad -k/v_P \\ 0 &{}\quad -d_T &{}\quad K_T/v_P\\ 0 &{}\quad 0 &{}\quad b \\ \end{array}\right) . \end{aligned}$$

The largest eigenvalue of *A* is *b*; hence the growth rate of the cell is $\mu =b$. The eigenvector corresponding to *b* has $T=K_TR/[v_P(b+d_T)]$, $P=[k/(v_pb)][\nu f_T/(b+d_T) - 1]R$. Since $P \ge 0$ we have $\nu f_T/(b+d_T) - 1 \ge 0$ or $a \ge b$. We now ask the following: what is the largest value of $\mu$ possible, and for what value of $f_R$ does that occur? Since $\mu =b = \rho f_R -d_R$, one may naively think that the largest possible value of $f_R$, namely $f_R=1$ will give the largest $\mu$. However, we also have the inequality $b \le a$; therefore, the largest value of $\mu$ occurs when $b=a$. This is the same conclusion as reached in the “Appendix” for the full PTR model under the large $m_T,m_R$ approximation. The condition $a=b$ immediately yields $f_R=f_\mathrm{max}$ with $f_\mathrm{max}$ given by (16), and $\mu = \mu _\mathrm{max}$ given by (17). Further the above eigenvector also reproduces (18) for *T*/*R*. In the linearized equation $\dot{P}=\mu P= K_PT-(k/v_P)R = (m_TT)\nu - (m_RR)\rho$, we can recognize the two terms as the outputs of the metabolic and ribosomal sectors.

The above approximation is reasonable for ${f_R} < {f_{\mathrm{max}}}$. It is meaningless for ${f_R} > {f_{\mathrm{max}}}$ because *P* turns negative in that regime under this approximation, though the full model has a perfectly reasonable behaviour even for ${f_R} >{f_{\mathrm{max}}}$. As seen earlier, this approximation is also good for deducing $f_\mathrm{max},{\mu _{\mathrm{max}}}$ and *T*/*R* as these tend to finite limits when $f_R$ approaches ${f_{\mathrm{max}}}$ from below. It is not useful for estimating *P*/*R* near ${f_R}={f_{\mathrm{max}}}$ which approaches zero in this approximation. One can see from the full model that *P*/*R* receives corrections in a small range of $f_R$ of size ${\sim }1/m_R$ around ${f_{\mathrm{max}}}$, in which range it goes from a value ${\gg }1$ to a smaller value. In the full model *P*/*R* does not go to zero at ${f_R} = {f_{\mathrm{max}}}$.

## Conclusion

In this paper we have constructed a simple dynamical system describing a cell in terms of its three coarse-grained molecular pools and shown that the optimization of the steady-state growth rate of the cell with respect to a parameter that can be tuned by intracellular regulation leads to the growth laws (1), (2) and (3). We have reproduced and extended existing formulae for the growth rate and other physiological parameters. This deepens our understanding of the macroscopic physiological variables in terms of microscopic parameters. We expect that this kind of model can be extended to include other molecular sectors in the cell (Hui et al. 2015).

At a methodological level we have introduced a scheme that allows an explicit computation of the steady-state growth rate of the cell in terms of the cellular and medium parameters. In this scheme a key assumption is that the volume of the cell is determined by its molecular populations. We have also put to use two natural large parameters in the cell, ${m_T}$ and ${m_R}$, to set the scale of certain other parameters and to make approximations. This has allowed us to get analytic results for the nonlinear system level dynamics.

Our model uses an optimization principle to fix an internal parameter, ${f_R}$, the fraction of ribosomes making ribosomes. The model is silent on the dynamical mechanisms inside the cell that implement this optimization. These mechanisms have been the subject of several recent works (Scott et al. 2014; Maitra and Dill 2015; Weiße et al. 2015; Bosdriesz et al. 2015). We hope that combining some of the methods introduced here with the mechanisms discussed in these works will produce models that are more satisfactory than the present one.

## Figures and Tables

**Fig. 3:**
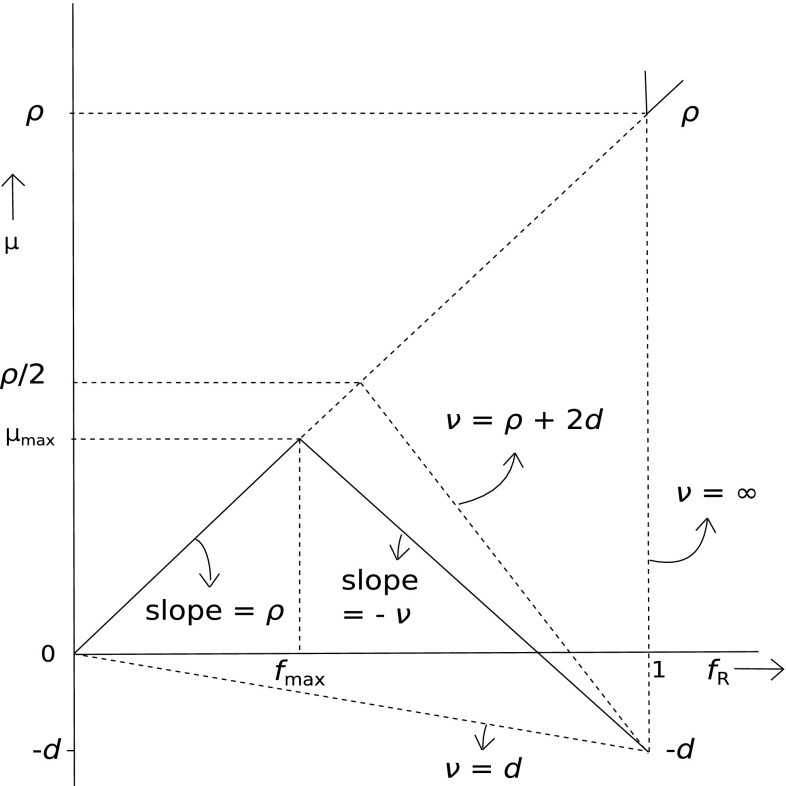
$\mu$ vs $f_R$ in the PTR model. The figure is illustrated for $d_R=0,d_T=d$. The *two* *solid lines* show $\mu$ as an increasing and then decreasing function of $f_R$, with a maximum $\mu _\mathrm{max}$ at $f_R=f_\mathrm{max}$. The *solid line* of slope $-\nu$ is shown for a generic value of $\nu$. The other *three dotted lines* with specific values of $\nu$ represent what this *solid line* would have been for those values of $\nu$. As $\nu$ increases from *d* to $\rho + 2d$ to $\infty$, $\mu _\mathrm{max}$ increases from 0 to $\rho /2$ to $\rho$

## Appendix

Under the large $m_T,m_R$ assumptions described in the main text, we ignore $\epsilon _1$ compared to 1 and $\epsilon _2$ compared to $a+b$ in (12). Thus

26 $$\begin{aligned} \alpha \approx 1,\quad \beta \approx a+b, \quad \gamma = ab, \end{aligned}$$

It follows that $\beta ^2 - 4\alpha \gamma$ is a perfect square;

27 $$\begin{aligned} \beta ^2 - 4\alpha \gamma =(a+b)^2-4ab =(a-b)^2. \end{aligned}$$

From Eq. (13) we have

28 $$\begin{aligned} \mu &= \frac{\beta - \sqrt{ \beta ^{2} - 4\alpha \gamma }}{2\alpha }={1 \over {2}}[(a+b)-|a-b|],\nonumber \\&= b \quad \mathrm{if} \; a \ge b,\nonumber \\&= a \quad \mathrm{if} \; a \le b. \end{aligned}$$

In the equation above $-\sqrt{ \beta ^{2} - 4\alpha \gamma }$ has been replaced by $-|a-b|$, because, as discussed earlier, it is this solution that has the correct physical behaviour. The point $a=b$ corresponds to $\nu f_T-d_T = \rho f_R - d_R$, or

29 $$\begin{aligned} f_R= {{\nu +d_R - d_T} \over {\nu +\rho }} = f_0. \end{aligned}$$

The region $a>b$ corresponds to $f_R<f_0$ and $a<b$ to $f_R>f_0$. Thus we have

30 $$\begin{aligned} \mu&= \rho f_R - d_R \quad \mathrm{for} \; f_R \le f_0,\nonumber \\ &= \nu - d_T - f_R \nu \quad \mathrm{for} \; f_R \ge f_0. \end{aligned}$$

Thus $\mu$ as a function of $f_R$ is given by the two straight lines of slope $\rho$ and $-\nu$ as shown in Fig.  3 (the solid lines). It is evident that the maximum value of $\mu$ is obtained where the two lines meet, which is at $f_R=f_0$. Using (29) this proves (16) in the main text. Further, from the first of Eqs. (30) it follows that $\mu _{\mathrm{max}} = \rho f_{\mathrm{max}} - d_R = [\rho (\nu - d_T) - \nu d_R]/(\nu + \rho )$. This proves (17).
